# Transcatheter Arterial Embolization Treatment for Bleeding Visceral Artery Pseudoaneurysms in Patients with Pancreatitis or following Pancreatic Surgery

**DOI:** 10.3390/cancers12102733

**Published:** 2020-09-23

**Authors:** Isao Numoto, Masakatsu Tsurusaki, Teruyoshi Oda, Yukinobu Yagyu, Kazunari Ishii, Takamichi Murakami

**Affiliations:** 1 Department of Radiology, Kindai University, Faculty of Medicine 377-2 Ohnohigashi, Osakasayama, Osaka 589-8511, Japan; numoto.isao@hotmail.co.jp (I.N.); teru.oda0321@gmail.com (T.O.); y-yagyu@med.kindai.ac.jp (Y.Y.); ishii@med.kindai.ac.jp (K.I.); 2 Department of Diagnostic Radiology, Kobe University, Graduate School of Medicine. 7-5-2, Kusunoki-Cho, Chuo-Ku, Kobe, Hyogo 650-0017, Japan; murataka@med.kobe-u.ac.jp

**Keywords:** embolization, postoperative hemorrhage, pancreatitis, pancreatic fistula, NBCA

## Abstract

**Simple Summary:**

Transcatheter arterial embolization (TAE) with coils is widely used to treat pseudoaneurysms; recently, the use of N-butyl cyanoacrylate (NBCA) in TAE has been reported as a feasible and effective approach. The purpose of our retrospective study was to evaluate the efficacy and safety of TAE with coils and NBCA for pseudoaneurysms associated with pancreatitis or pancreatic surgery. This retrospective study included 42 consecutive patients. The technical and clinical success rates, incidence of recurrent bleeding, complications, including pancreatitis, and overall survival after TAE were evaluated. All cases obtained hemostasis after TAE (the technical success rate was 100%). Complications were seen in only two patients. Clinical success rate that was evaluated in terms of 30-day mortality was 76.2%. TAE is then an effective treatment modality for pseudoaneurysms associated with pancreatitis or pancreatic surgery. Accurate diagnosis using angiography contributes to the proper choice of embolic agents and management of such hemorrhages.

**Abstract:**

Purpose: To evaluate the efficacy and safety of transcatheter arterial embolization (TAE) for pseudoaneurysms occurring secondary to pancreatitis or because of leakage of pancreatic juice after pancreatectomy. Materials and Methods: This retrospective study included 42 consecutive patients (38 males and 4 females; mean age, 60 years; range, 33–80 years) who underwent TAE for bleeding visceral artery pseudoaneurysms between March 2004 and December 2018. The technical and clinical success rates, incidence of recurrent bleeding, complications, including pancreatitis, and overall survival after TAE were evaluated. Results: Of the 42 enrolled patients, 23 had bleeding due to a complication of pancreatectomy and 19 had bleeding as a complication of pancreatitis. TAE with N-butyl cyanoacrylate (NBCA) or NBCA plus microcoils recurrent bleeding or inability to control bleeding was 15.8% (3 of 19) following TAE with NBCA and 17.4% (4 of 23) following TAE with coils. No clinically significant ischemic events of the pancreas or duodenum were observed in the embolized areas. Serum amylase did not increase compared with the initial levels after any of the procedures. At 30 days after TAE, 32 patients were alive. Conclusion: TAE has a high success rate for the management of hemorrhage, with few complications. The procedure appears to be safe and effective for pseudoaneurysms associated with either pancreatitis or pancreatectomy.

## 1. Introduction

The occurrence of pseudoaneurysms associated with pancreatitis or pancreatic surgery is a rare but life-threatening complication [1,2]. Massive hemorrhage from a pseudoaneurysm has been recognized as the most rapidly lethal complication of pancreatic surgery/pancreatitis, with a reported mortality rate of 25–50% [1,2]. Transcatheter arterial embolization (TAE) with coils is widely used to treat pseudoaneurysms; recently, the use of N-butyl cyanoacrylate (NBCA) in TAE has been reported as a feasible and effective approach [3]. Few studies have shown the benefits of NBCA and few have examined long-term outcomes. The present retrospective study aimed to evaluate the efficacy, safety and long-term outcomes of TAE using not only coils but also NBCA for pseudoaneurysms that occurred secondary to pancreatitis or because of pancreatic juice leakage at the site of pancreatectomy.

## 2. Results

### 2.1. Study Population

Of the 42 patients enrolled in this study, the majority were male, and the mean age was 60 (range, 33–80) years. Data on the type of pancreatitis, pancreatic surgeries performed, and signs of hemorrhage identified are summarized in Table 1. Nineteen patients underwent TAE with NBCA: nine underwent the procedure for postoperative hemorrhage and 10 for bleeding secondary to pancreatitis. Twenty-three patients underwent TAE with coils: 14 underwent the procedure for postoperative hemorrhage and nine for bleeding secondary to pancreatitis. All TAEs were technically successful, and immediate hemostasis was achieved. Clinical success was 32/42 (76.2%). Clinical success tended to be higher with NBCA than with coils, but there were no significant differences between NBCA and coils (odds ratio = 2.2, *p* value = 0.305) by fisher’s exact test. The outcomes are summarized in Table 2.

Continuous data are presented as mean (range), categorical data are presented as number. Of the pancreatitis, acute pancreatitis and chronic pancreatitis were present in 10 (23.8%) and 9 (21.4%) cases, respectively. Of the surgery, pancreatectomy for malignant tumor, Frey’s procedure for chronic pancreatitis, necrosectomy for acute pancreatitis and total pancreatectomy for chronic pancreatitis were present in 15 (35.7%), 4 (9.5%), 3 (7.2%) and 1 (2.4%) cases, respectively.

### 2.2. Recurrent Bleeding

Recurrent bleeding occurred in four patients after embolization with a coil and in three patients after embolization with NBCA. Of those with embolization with NBCA, one patient underwent re-TAE for recurrent bleeding. In this case, the first procedure was performed using NBCA admixed with iodized oil at a ratio of 1:3. After recurrent bleeding occurred, TAE with NBCA admixed with iodized oil at a ratio of 1:2 was performed. Recurrent bleeding did not occur after the second intervention. One patient underwent angiography due to decreased hemoglobin; however, the sites of bleeding were not found, and the patient died of hypovolemic shock. The other patient with recurrent bleeding after embolization with NBCA underwent embolization due to pseudoaneurysm associated with pancreatitis, and this patient underwent Frey’s procedure two months after the first TAE. This patient had melena one month after the surgery. The bleeding site was the posterior superior pancreaticoduodenal artery, which was different from the bleeding site after the first TAE. The patient underwent re-TAE with NBCA, which stopped the bleeding. Of the patients who underwent embolization with a coil and suffered recurrent bleeding, two underwent repeat diagnostic angiography; however, the bleeding sites were not found and both patients died of shock. One received no treatment for recurrent bleeding and died of hypovolemic shock. In the other patient with recurrent bleeding after coil embolization, the hemorrhage could not be controlled and pancreatectomy was performed four days after TAE. This was the only case of rebleeding in which surgical hemostasis was performed. 

### 2.3. Complications

Major complications occurred in two patients who underwent TAE with a coil. One patient suffered a hepatic abscess 14 days after proper hepatic artery embolization, and the other had gastrointestinal perforation 20 days after celiac artery embolization. No patients had pancreatitis after TAE. Seven patients died within 30 days after TAE with coils: five due to multiple organ failure and two due to hypovolemic shock after recurrent bleeding. Three patients with NBCA died within 30 days: one due to multiple organ failure, one due to hypovolemic shock after recurrent bleeding, and one due to septic shock and disseminated intravascular coagulation after developing a gastrointestinal perforation.

### 2.4. Number of Hospitalization Days after TAE and Overall Survival

**Figure 1:**
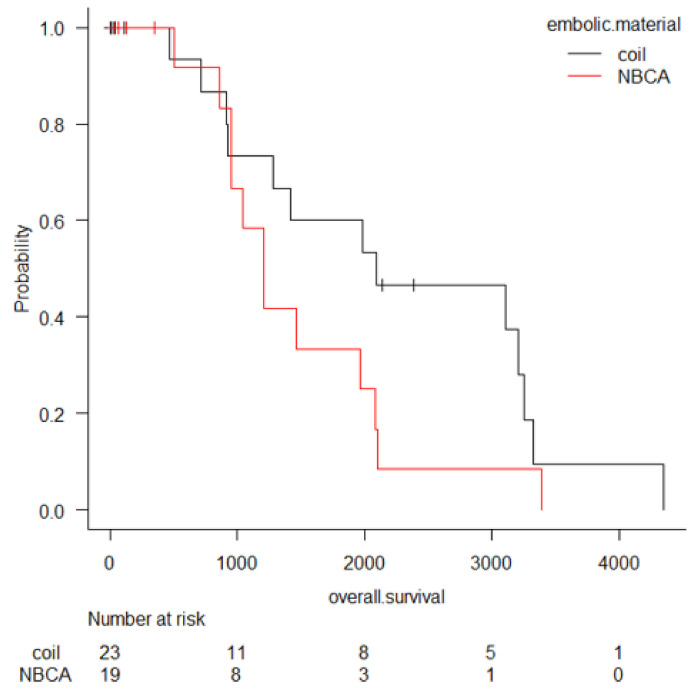
Overall survival after transcatheter arterial embolization (TAE) for hemorrhage due to pancreatitis or pancreatectomy. Patients who underwent TAE with coils tend to survive longer than those who underwent TAE with NBCA, however there is no significant difference.

The overall survival was 1–4349 days (median: 1202 days), including 17 deaths. The Kaplan Meyer curve is shown in Figure 1. The overall survival of recurrent bleeding cases (median: 2082 days) and others (median: 1438 days) was compared, but there was no significant difference (*p* value = 0.85) by log-rank test. The overall survival of rebleeding cases (median: 961 days) and others (median: 1964 days) was compared, but there was no significant difference (*p* value = 0.07) by log-rank test.

## 3. Discussion

Pseudoaneurysms can occur due to both acute and chronic pancreatitis. Leakage of pancreatic juice can cause enzymatic degradation of the adjacent arterial wall and subsequent rupture [4]. It is believed that in the case of pseudoaneurysms associated with pancreatectomy, extensive skeletonization of the artery may injure the vessel wall. Furthermore, postoperative leakage from the pancreatic jejunostomy or hepaticojejunostomy may result in the digestion of vascular structures by erosive pancreatic or biliary juice, respectively. In addition, subsequent abscess formation can erode the vessel wall or vascular anastomosis [5,6]. Some studies have shown the mortality rate due to pseudoaneurysms associated with pancreatitis to be lower than that due to pseudoaneurysms occurring after surgery [1,7,8,9,10]. Zyromski et al. compared pseudoaneurysms associated with pancreatitis and those that arose after pancreatic surgery and found pseudoaneurysms that occurred due to pancreatitis to be associated with significantly lower mortality rates [11]. Other studies have reported the recurrent-bleeding rate after embolization for pancreatitis to be 0–21% [12,13,14] and that after surgery to be 0–50% [6,15]. Our results were similar to the results of those studies. The recurrent-bleeding rate after TAE for pseudoaneurysms associated with pancreatectomy (33%) was higher than that observed in pancreatitis (7%). Moreover, the present study showed that post-pancreatectomy hemorrhages were more severe than hemorrhages associated with pancreatitis in terms of clinical outcomes and complications. 

Coils are appropriate embolic materials for TAE in the case of pseudoaneurysms, and coil embolization is an effective approach for the management of hemorrhages [1,7,16,17]. The coil needs to be placed in both the proximal and distal arteries of the pseudoaneurysm, and it is sometimes difficult to select the distal artery in small or severely affected vessels. Liquid embolic agents such as NBCA have low viscosity and so can be easily injected through small or tortuous vessels either upstream or downstream of the pseudoaneurysm, as a single injection. In addition, the mechanism underlying the effects of NBCA does not depend on the coagulation process; therefore, the drug can be used in patients with complicated coagulopathies [18]. However, NBCA can cause serious complications including liver infarction, duodenal infarction, and pancreatitis [7]. Mixtures of NBCA are difficult to handle, and the viscosity of the liquid contrast medium is different from that of NBCA. Therefore, considerable experience is required to achieve optimal results.

In this study, two complications occurred following TAE with coils, although there were no significant differences between the complications after TAE with the use of coils and those after embolization with NBCA. Coils tend to be used to embolize large vessels such as the common hepatic artery. Not all collateral tracts are eliminated, but in some cases, surgical procedures reduce some collateral tracts. In this condition, TAE have the risk of complications such as hepatic abscesses secondary to hepatic infarction. It is possible that ligation of collateral branches that could replace hepatic vascularization might have occurred during pancreatic surgery; therefore, it is essential to ensure that the portal venous system is patent before embolization of the hepatic artery.

In the present study, hemostasis was achieved in two of the three cases of recurrent bleeding after TAE with NBCA; however, bleeding could not be stopped with or without re-TAE in any of the cases of recurrent bleeding after TAE with coils. Yamakado et al. reported successful hemostasis in three cases of recurrent bleeding after initial TAE with coils, which was achieved by TAE with NBCA [19]. The results of these and the present study therefore indicate that TAE with NBCA may be an effective method for managing recurrent bleeding.

When hemorrhage was controlled after TAE, long-term survival without complications could be achieved and long-term survival could also be achieved in cases of controlled recurrent bleeding and complications; however, death occurred in some cases (eight) due to multiple organ failure and/or sepsis. These cases were associated with severe infected pancreatic or biliary juice or walled-off necrosis (WON). Infection in necrotic tissues leads to sepsis and multiple organ failure. It is important to treat pancreatic or biliary juice or WON not only before TAE to prevent hemorrhage, but also after TAE to prevent recurrent bleeding and organ failure. If controlling infection is difficult only by internal medicine, intervention such as endoscopic, or percutaneous drainage, or endoscopic or surgical necrosectomy should be considered. Patients who underwent TAE with coils tend to survive longer than those who underwent TAE with NBCA. This might be one of the causes that NBCA was selected in cases of a severe coagulation status following disseminated intravascular coagulation (DIC) due to severe infection. However, the overall survival time is mainly affected by the patient’s background.

The effectiveness of covered stent embolization of the hepatic and superior mesenteric arteries has been reported [20,21]. A covered stent makes it possible to attain effective hemostasis and preserve vascular flow, and usually results in less hepatic complications than hepatic artery occlusion. However, covered stents such as Viabahn stent-graft (W.L. Gore & Associates, Flagstaff, AZ, USA) had not been adopted in Japan for embolization for hemostasis prior to June 2017. A covered stent was used in combination with coils in three cases, and hepatic artery and superior mesenteric artery flow was prevented in all cases. Therefore, covered stents may be useful for hemostasis in large vessels such as the celiac, common hepatic, and superior mesenteric arteries.

Our study had several limitations that should be acknowledged. First, this was a single-center retrospective study. Second, the safety and efficacy of TAE could not be compared with those of reoperation for postoperative hemorrhage because TAE is the recommended therapeutic strategy at the study institution. Third, this study had selection bias regarding the choice of embolic material. Main trunk embolization might have higher potential of complications such as hepatic abscess; main trunk embolization tends to use coils, and therefore the complication rate tends to be higher than that with NBCA embolization.

## 4. Materials and Methods

### 4.1. Patients

This retrospective study was approved by the appropriate institutional review board, and the need for written informed consent was waived. There were 45 consecutive patients treated for signs of bleeding from pancreatitis or following pancreatic surgery between March 2004 and December 2018. During this period, three cases of surgical hemostasis were performed in our hospital. Two of them were bleeding from main portal vein or collateral vein. The other was due to hemorrhage accompanied by anastomotic dehiscence of pancreaticogastrostomy. The remaining 42 patients (38 males and 4 females) who underwent TAE for bleeding visceral artery pseudoaneurysms were recruited. Data on patients’ demographics, clinical presentation, and medical history, as well as computed tomography (CT) and angiographic findings, were collected. A pseudoaneurysm was diagnosed as an aneurysm that occurred after pancreatitis or pancreatectomy within a short time.

### 4.2. Embolization

**Figure 2:**
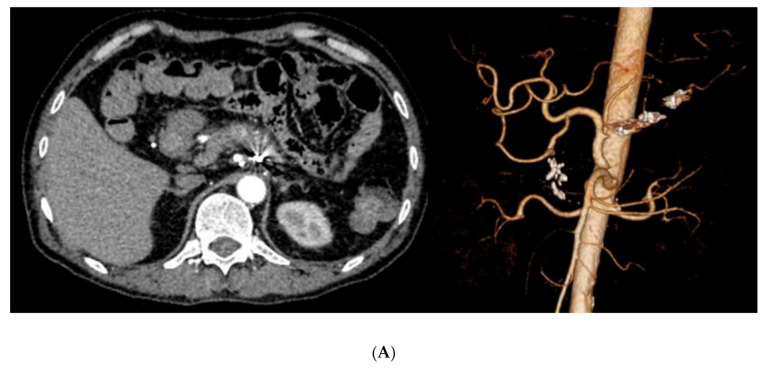

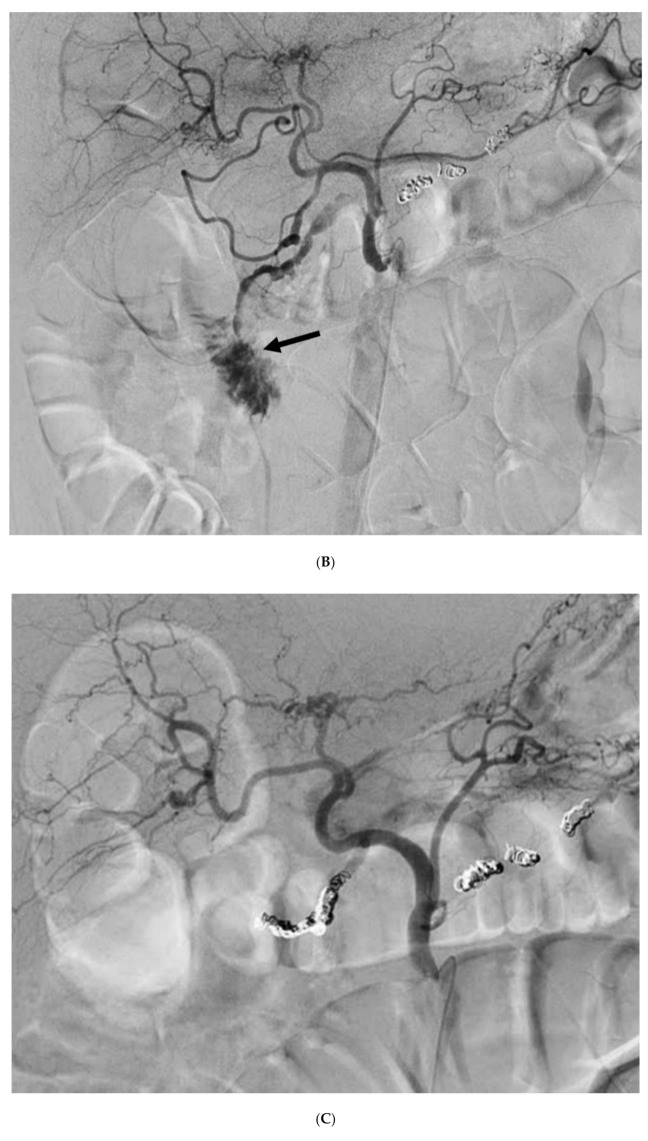
Transcatheter arterial embolization with coils. Angiographic images of a 55-year-old man with chronic pancreatitis. The patient had melena and endoscopic hemostasis was attempted, but the bleeding could not be stopped. (**A**) Contrast-enhanced CT was performed but a pseudoaneurysm was unclear by contrast-enhanced and three-dimensional CT. (**B**) The celiac axis angiogram revealed extravasation (indicated by the arrow) from the gastroduodenal artery. A microcatheter could be inserted into the artery over the pseudoaneurysm, and coils were placed between the distal and proximal sites of hemorrhage. (**C**) After embolization with a coil, hemostasis was obtained. The patient had no signs of recurrent bleeding or complications after transcatheter arterial embolization.

**Figure 3:**
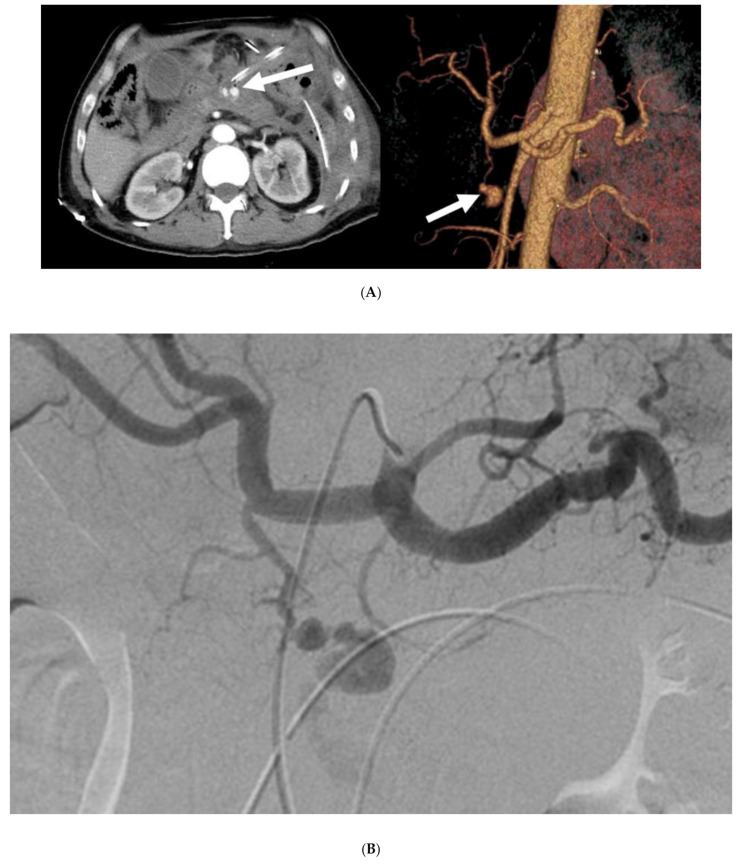

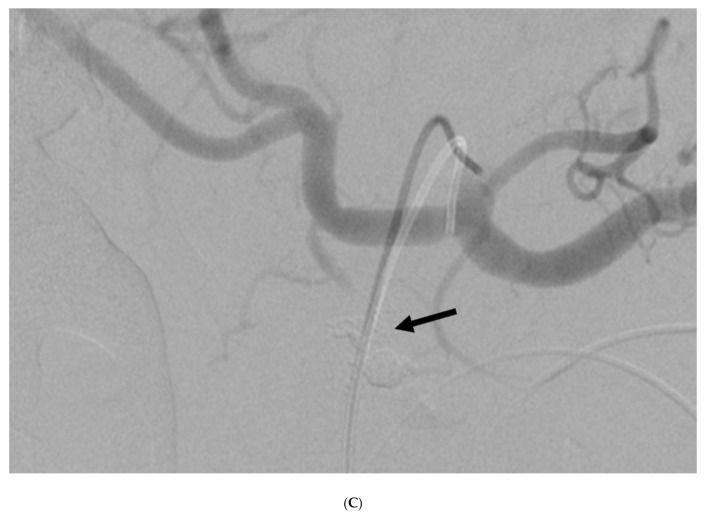
Transcatheter arterial embolization with N-butyl cyanoacrylate. Angiographic images of a 73-year-old man who underwent distal pancreatectomy for pancreatic body cancer. Bloody fluid was detected in the drainage tube 13 days after surgery, and contrast-enhanced computed tomography was performed. (**A**) A pseudoaneurysm (indicated by the arrows) was identified in the gastroduodenal artery by contrast-enhanced and three-dimensional CT (left and right images, respectively). (**B**) The celiac axis angiogram revealed a pseudoaneurysm in the gastroduodenal artery. A microcatheter could not be inserted into the distal site of bleeding, and so 0.6 mL of a 1:3 mixture of N-butyl cyanoacrylate/iodized oil was injected from the proximal site of hemorrhage. (**C**) Hemostasis was achieved after embolization with N-butyl cyanoacrylate (indicated by the arrow). There were no signs of recurrent bleeding or complications after transcatheter arterial embolization.

Diagnostic angiography was performed to identify the bleeding site using a 4-F catheter and 1.7–2.1-F microcatheters. Portography results were evaluated using portal-phase images of the celiac or superior mesenteric arteriographies. In all patients, a 4-F catheter was used to access the suspected site of hemorrhage. Next, a microcatheter was inserted as close as possible to the bleeding site. When the microcatheter could be inserted into the distal artery, microcoils were usually used (Figure 2). Therefore, coils were mostly used in the main trunk. Embolization using coils was performed with the isolation technique. Embolization using NBCA was performed in cases where the microcatheter could not be inserted into the distal artery. Therefore, NBCA tends to be used in branches. Moreover, the patient who had coagulopathy due to substantial blood loss underwent embolization using NBCA. If blood flow was rapid before NBCA embolization, coils were placed to decrease the blood flow in order to prevent the influx of NBCA into the organs and to obtain good penetration through the pseudoaneurysm (Figure 3). When NBCA was used for TAE, because of its lack of radiopacity and rapid polymerization, iodized oil was admixed (NBCA mixture), and the ratio of NBCA to iodized oil was modified to alter the rate of polymerization and control the degree of distal vessel penetration [22]. A ratio of 1:2–1:6 of NBCA to iodized oil was used. Test injections with contrast medium were performed prior to injection of the NBCA mixture to identify the appropriate dose. The inner lumen of the microcatheter was filled with a glucose solution to prevent contact between the blood and NBCA during injection, and then the NBCA mixture was continuously injected. The microcatheter was quickly removed immediately after the administration of NBCA. Embolization was considered successful when the blood flow in the target vessel ceased. When embolization was not successful, the same procedure was repeated until afferent arteries were eliminated.

### 4.3. Sites of Pseudoaneurysms

The sites of pseudoaneurysms are detailed in Table 3. In cases of hemorrhage associated with pancreatitis where there was no bleeding after surgery, the splenic artery was the most common bleeding site; however, none of the patients who underwent NBCA embolization had bleeding from the splenic artery. Following NBCA embolization, the gastroduodenal artery was the most common bleeding site, which was also the case after pancreatic surgery. There were only two cases of bleeding from the gastroduodenal artery after pancreatitis. The clinical findings are summarized in Table 1.

### 4.4. Outcomes

The rates of technical and clinical success, recurrent bleeding, complications including pancreatitis, and overall survival after TAE were evaluated. Technical success was defined as embolization of a focal lesion or intentional reduction/cessation of blood flow to a target vascular bed or an entire organ. Recurrent bleeding was identified by signs of bleeding such as hematemesis, melena, bloody fluid in the drainage tube, or decreasing hemoglobin levels during 30 days of follow-up. Complications were defined as prolonged hospitalization to treat for complications and permanent adverse sequelae. Pancreatitis was defined as increasing levels of serum amylase after the procedures. Clinical success was evaluated in terms of 30-day mortality. Statistical comparisons with Fisher’s exact test were performed to determine the difference between the NBCA and coils. A *p* value of less than 0.05 was considered statistically significant.

## 5. Conclusions

In Conclusion, TAE is an effective treatment modality for hemorrhage associated with pancreatitis or pancreatic surgery. Accurate diagnosis using angiography contributes to the proper choice of embolic agents and management of such hemorrhages.

## Figures and Tables

**Table 1:** Study population characteristics.

Variable	Total Patients (*n* = 42)
Age, years	60.17 (33–80)
Gender	
Male/Female	38 (90.5%)/4(9.5%)
Cause of bleeding	
Pancreatitis/Surgery	19 (45.2%)/23 (54.8%)
Symptoms of hemorrhage	
Abdominal pain	14 (33.3%)
Bloody fluid from drainage tube	17 (40.5%)
Gastrointestinal hemorrhage	8 (19.0%)
None	3 (7.2%)

**Table 2:** Summary of outcomes.

Result		NBCA	Coils	Total	*p* Value
Technical success (%)		19/19 (100)	23/23 (100)	42/42 (100)	1.0
					
Recurrent bleeding (%)		3/19 (15.8)	4/23 (17.4)	7/42 (16.7)	1.0
	Post-surgery	2/9 (22)	4/14 (28.6)	6/23 (26.1)	
	Pancreatitis	1/10 (10)	0/9 (0)	1/19 (5.3)	
					
Complications (%)		0/19 (0)	2/23 (8.7)	2/42 (4.8)	0.238
	Post-surgery	0/9 (0)	2/14 (14.3)	2/23 (8.7)	
	Pancreatitis	0/10 (0)	0/9 (0)	0/19 (0)	
					
Pancreatitis (%)		0/19 (0)	0/23 (0)	0/42 (0)	
					
Clinical success (%)		16/19 (84.2)	16/23 (69.6)	32/42 (76.2)	0.305
	Post-surgery	7/9 (77.8)	7/14 (50)	14/23 (60.1)	
	Pancreatitis	9/10 (90)	9/9 (100)	18/19 (94.7)	

Data are presented as numbers. Abbreviations: NBCA, N-butyl cyanoacrylate.

**Table 3:** Distribution of artery origins of pseudoaneurysms.

Bleeding Artery	Post-Surgery	Pancreatitis	Total
Celiac artery	2 (4.8%)	0 (0%)	2 (4.8%)
Common hepatic artery	4 (9.5%)	0 (0%)	4 (9.5%)
Proper hepatic artery	1 (2.4%)	0 (0%)	1 (2.4%)
Splenic artery	0 (0%)	10 (23.8%)	10 (23.8%)
Dorsal pancreatic artery	2 (4.8%)	1 (2.4%)	3 (7.2%)
Gastroduodenal artery	8 (19%)	2 (4.8%)	10 (23.8%)
Pancreaticoduodenal arcade	4 (9.5%)	3 (7.2%)	7 (16.7%)
Gastric artery	0 (0%)	2 (4.8%)	2 (4.8%)
Superior mesenteric artery	2 (4.8%)	0 (0%)	2 (4.8%)
Middle colic artery	1 (2.4%)	0 (0%)	1 (2.4%)

Data are presented as numbers.
