# Correction: Isao N., et al. Transcatheter Arterial Embolization Treatment for Bleeding Visceral Artery Pseudoaneurysms in Patients with Pancreatitis or Following Pancreatic Surgery. *Cancers* 2020, *12*, 2733

**DOI:** 10.3390/cancers13061312

**Published:** 2021-03-15

**Authors:** Isao Numoto, Masakatsu Tsurusaki, Teruyoshi Oda, Yukinobu Yagyu, Kazunari Ishii, Takamichi Murakami

**Affiliations:** 1 Department of Radiology, Faculty of Medicine, Kindai University, 377-2 Ohnohigashi, Osakasayama, Osaka 589-8511, Japan; numoto.isao@hotmail.co.jp (I.N.); teru.oda0321@gmail.com (T.O.); y-yagyu@med.kindai.ac.jp (Y.Y.); ishii@med.kindai.ac.jp (K.I.); 2 Department of Diagnostic Radiology, Graduate School of Medicine, Kobe University, 7-5-2, Kusunoki-Cho, Chuo-Ku, Kobe, Hyogo 650-0017, Japan; murataka@med.kobe-u.ac.jp

The authors are sorry to report that the overall survival reported in their recently published paper was incorrect [1]. The results of overall survival had too many confounding factors to be appropriate for comparison. Consequently, the authors wish to make the following corrections to the paper:

The sentence mentioning overall survival after TAE should be deleted in the simple summary and abstract: “The technical and clinical success rates, incidence of recurrent bleeding, complications, including pancreatitis, and overall survival after TAE were evaluated.”

The Subsection "2.4. Number of Hospitalization Days after TAE and Overall Survival", including Figure 1, should be deleted:

*2.4. Number of Hospitalization Days after TAE and Overall Survival*

The overall survival was 1–4349 days (median: 1202 days), including 17 deaths. The Kaplan Meyer curve is shown in Figure 1. The overall survival of recurrent bleeding cases (median: 2082 days) and others (median: 1438 days) was compared, but there was no significant difference (*p* value = 0.85) by log-rank test. The overall survival of rebleeding cases (median: 961 days) and others (median: 1964 days) was compared, but there was no significant difference (*p* value = 0.07) by log-rank test.

Consequently, the original Figures 2 and 3 should be numbered as Figures 1 and 2 respectively.

The following sentences in the fifth paragraph in the Discussion Section should be deleted: “Patients who underwent TAE with coils tend to survive longer than those who underwent TAE with NBCA. This might be one of the causes that NBCA was selected in cases of a severe coagulation status following disseminated intravascular coagulation (DIC) due to severe infection. However, the overall survival time is mainly affected by the patient’s background.” 

The first sentence in Section 4.4. should be revised from “The rates of technical and clinical success, recurrent bleeding, complications including pancreatitis, and overall survival after TAE were evaluated.” to “The rates of technical and clinical success, recurrent bleeding and complications including pancreatitis, were evaluated”.

Additionally, the original affiliation 1 should be changed from “Department of Radiology, Kindai University, Faculty of Medicine” to “Department of Radiology, Faculty of Medicine, Kindai University”. The original affiliation 2 should be changed from “Department of Diagnostic Radiology, Kobe University, Graduate School of Medicine” to “Department of Diagnostic Radiology, Graduate School of Medicine, Kobe University”.

The authors apologize for any inconvenience caused and state that the scientific conclusions are unaffected. The original article has been updated. 

## Figures and Tables

**Figure 1:**
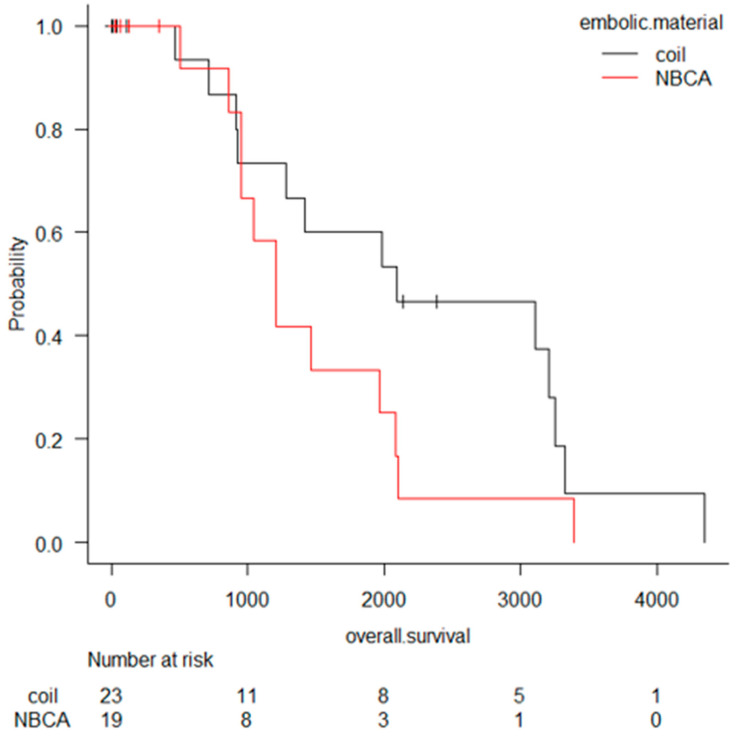
Overall survival after transcatheter arterial embolization (TAE) for hemorrhage due to pancreatitis or pancreatectomy. Patients who underwent TAE with coils tend to survive longer than those who underwent TAE with NBCA, however there is no significant difference.
